# Observations on the Deaf and Dumb

**Published:** 1858-11

**Authors:** Richard J. Dunglison

**Affiliations:** Philadelphia


### Art I.—*Observations on the Deaf and Dumb.*—By Richard J. Dunglison, M.D., of Philadelphia.

There are numerous subjects of deep interest, connected with the study of medicine, which are overlooked by a large majority of the profession or are passed by as undeserving of any special notice. It is to be regretted that medical men have at all times been so intently occupied in the search after curative agents, with which to combat disease, that they have failed to pay that attention to collateral subjects which they so well merit. Defeated, at times, in the acquisition of the knowledge which they covet, they have neglected to study the malady which they have found incurable; and, in the belief that no practical good could result to mankind from a continued investigation of such affections, they have often left the unfortunate sufferers to the merciful care of the few who might have more charitable views in regard to them.

It would be unjust to the present enlightened generation to charge upon them the possession of such sentiments to as great an extent as their predecessors; and yet we cannot close our eyes to the fact, that the attainment of a practical education excludes, too often, any thought of subjects which should awaken the liveliest sympathies of the physician. Why is it that medical literature has so long been deficient in works upon the deaf and dumb; and why have facts of statistical or social interest, bearing upon the subject, been so rarely supplied to the profession and the world? It cannot be supposed that it was due to the existence of a smaller proportionate number of that class formerly than exists at the present day; for the rate of increase or diminution is so insignificant for a series of years, that, on some points, we can speak of the past with almost as much certainty as the present. In regard to the medical men of days past, may we not rather assign as a sufficient cause for the absence of any satisfactory information in regard to deaf-mutes, the consciousness of an utter ignorance of all means tending to their relief?

The objection urged at times against the study of special diseases weighs but little as compared with the importance of acquiring and disseminating information upon subjects of such vital consequence as we find embraced in the too often incurable affections of the senses. Devolving, as it does, upon the medical profession to diffuse information upon points of anthropology, which cannot be generally known except by study, and careful, thorough examination of medical facts and reasonings, how necessary must it be that all the data that can be collected should be arranged in some tangible form, and all the general laws upon special subjects should be classified!

We are now, so to speak, only at the commencement of the task; we see the errors of past records or statistics; we endeavor to sift out from the confused mass that which may be serviceable as a foundation on which to base our present and future investigations; and we look forward to the confirmation or destruction of our present deductions according as future observations may influence them.

If it were asked which form of deprivation of the organs of sense has the most claim upon our attention and sympathy, it would be difficult to decide. The deaf-dumb and the blind present two distinct classes, affording many points of interest as regards their influence upon society, and the reactions of social influences upon them; and both seem to be governed throughout the world by general laws. The present article includes only the consideration of that class of human beings who, born with the infirmity existing, from causes whose nature we shall hereafter examine, or acquiring it at a subsequent period, suffer from the deprivation of two important means of communication with the external world, viz. the faculties of audition and speech.

It is a remarkable fact that accurate statistics of the deaf and dumb are, even now, not easily attainable; few comprehensive works have been issued that embrace the full details of the past and present condition of this interesting class of unfortunates,—their proportionate numbers in different countries, and the history of their various modes of instruction. It is true that the annual reports of institutions sometimes contain valuable information; but, in a large number of cases, too much space is given to details affecting merely local interests, and too little to topics of universal consequence. The task, therefore, of collecting, from so many different channels, information that may be of practical value, is by no means easy; and much labor is required to sift out points of real importance from the mass of materials presented.

Even those works which have devoted the most attention to the subject, and have exhibited evidences of profound research and a desire to do the matter full justice, have, for reasons which will be hereafter referred to, fallen short of their object, and failed to convey an accurate statistical estimate of the actual condition of the deaf and dumb. This is to be regretted, not only because it impairs the value of investigations undertaken in the most laudable spirit of philanthropic inquiry, but also exerts an influence at the present day, in partially paralyzing our attempts at the formation of comparative tables of the past and present. Yet, in spite of difficulties, the multitudes of scattered facts are being classified, general laws are deduced from reliable data, and more and more careful regard will doubtless be paid to the proper method of forming accurate statistical tables.

There are many points in connection with an enumeration of the deaf and dumb, and, indeed, in censuses of all descriptions, which, although viewed often as matters of secondary importance by those who scan only the ordinary details of population, etc., are to the medical profession subjects of eminent moment. The influence of sex, of race, and of locality, in predisposing to various maladies, is deducible from careful comparisons of statistical tables, furnished from various sources and at different periods. Several of these points are not often fully discussed, and to them I desire to draw some attention in the course of the present article. Those on which I would wish particularly to dwell are—

1. The apparently greater prevalence of deaf-mutism in countries which have mountainous ranges, and the small ratio of the deaf and dumb to the population in low countries.

2. The influence of race in predisposing to deaf-mutism, as exemplified in our own country more particularly.

3. The duration of life among the deaf and dumb, and the age of greater prevalence of deaf-mutism.

4. Deaf-dumbness in the United States, and institutions for the instruction of deaf-mutes.

5. An estimate of the number of the deaf and dumb in the known world.

But, before entering upon any of these points, it is worthy of note, as an evidence of the existence of a general law governing, so far as we know, a great portion of the human family, that there is little or no variation in the proportionate numbers of deaf-mutes in the same locality, even in large tracts of country, during successive periods of time. This rule does not apply alone to the class under consideration; it seems to be applicable to varied conditions of health and disease in all parts of the globe. Yet it yields, at times, to the ravages of desolating epidemics, which seem to put all human laws at defiance, bringing in their train consequences that frequently produce a most marked increase in the ratio of the deaf and dumb or blind to the general population. The influence of epidemic malignant scarlatina, as an agent of this class, will be referred to in the consideration of the causes of acquired deaf-dumbness.

1. That there is a greater aggregate of deaf-mutism in mountainous regions seems, as a general rule, to be established by statistical returns from almost all countries in which statements of the condition of the deaf and dumb have been published. It is remarkable that the same causes, which appear to be at work in certain localities to produce idiocy, are, apparently, active as predisposing agents in the propagation of deaf-mutism. And yet we must confess our utter ignorance of any precise connection between them. These effects manifestly, however, evince the existence of similar local causes of arrest of development. Yet the number of blind in the same localities is not above the ordinary average. Are there influences that affect the one set of organs and not the other; and, if so, what do we know of the atmospheric phenomena, or of the special geographical nature of the country, or, indeed, peculiarities of any kind that will rationally solve the question?

These are problems not easily explained, requiring a prolonged examination of facts, and an intimate knowledge of localities; a combination of requisites for the task, which may some day enlighten us in regard to certain of the predisposing and exciting causes of deaf-mutism. But, at present, we are very much in the dark; we know that such influences are productive of such results, and yet we have failed thus far satisfactorily to account for them. To show that there is strong ground for our belief that locality has a claim upon our attention, as one of the causes of deaf-mutism, statistical returns from the various countries of Europe may be referred to with advantage. One of the latest published statements gives the average proportion of deaf-mutes to the population in Europe as 1 : 1593, or about one in every 1600 inhabitants.

Switzerland is a remarkable exemplification of the prevalence of deaf-dumbness in certain localities. It is here, as is well known, that cretinism, that scourge of certain districts of the Swiss country, prevails so extensively; and in those regions which suffer the most from its ravages deaf-mutism is of very common occurrence. It would be interesting to know—and future statisticians may throw light upon the subject—whether cretinism and deaf-dumbness are often met with in the same individual, and whether the one is of much more frequent occurrence than the other. Returns from several of the Cantons, taken about thirty years ago, give the following result:—

| | |
|---|---|
| Population of five Cantons . . . . . . . | 895,000 |
| Number of deaf-mutes . . . . . . . . | 1777 |
| Proportion to population . . . . . . . . | 1 in 503 |

Later and more reliable statements give even a greater proportion than this; for we learn from tables prepared within the past few years, in regard to the population, etc. of the mountain passes of Switzerland, that the proportion is often as great as one in 206.

More striking facts than these, however, are contained in the "*Journal de l'Instruction des Sourds-Muets,*" in regard to the Canton of Vaud. In sixty-seven parishes there is said to be not a single deaf-mute; while in the remaining fifty-five there are as many as 152, or a proportion of one to every 100 of the population. What may be the cause that opposes the prevalence of deaf-dumbness in the former, it is impossible to say; but in the latter we have people living in deep valleys, surrounded on almost every side by mountains, and with a difficulty of access from neighboring parishes or Cantons, that might be sufficient cause for marriages of consanguinity to be of frequent occurrence.

The English census furnishes information from the various countries of Great Britain, viz., England, Scotland, and Wales; the Irish census being a separate feature, and much more accurate than the English. Little regard had been paid to statistics of the deaf and dumb in these countries, until a general desire for exact information stimulated the proper authorities to make inquiries on the subject, the results of which are comprised in the census of 1851. We can now, therefore, speak from actual and comparative facts, instead of conjectural statements founded on the numerical estimates of other countries. Here we find the greatest prevalence of deaf-mutism in the Northern counties of Scotland, a district of country remarkable for its mountainous ranges, and whose valleys even are far above the level of the Southern lowlands. There seems to be an exception to our rule in the Northern counties of England, for there the proportionate number of deaf-mutes is small. Cumberland, for example, one of the most mountainous districts of England, has a proportion of one to 1916 of its inhabitants, which is far less than its mountainous aspect might induce us to suppose. The test, however, is so satisfactory for almost all the counties of Great Britain, that we cannot but imagine that some local causes, of the exact nature of which we are ignorant, may operate against it in Westmoreland and Cumberland.

In Ireland we find a proportion of one to every 1380 of the population. Sources of error have been pointed out by one of the Commissioners of the Census, (Mr. Wilde,) and he is disposed to infer that the ratio really is about one to 1579, a proportion which corresponds very closely with that of Europe generally, and may be a reliable basis for our calculations. The point of interest, however, is in regard to the physical appearance of the country; and in this Ireland is not an exception to the general rule, the inhabitants of counties which present mountain ranges being, almost without exception, more affected with deaf-dumbness. In the open country, where the surface is level, the proportion is sometimes as small as one in 2000; but in hilly situations, such as Limerick and Tipperary, it is as much as one in 1300; and in Wicklow, which is one of the most mountainous counties in Ireland, one in 1192.

The number of deaf-mutes in the Alpine and Carpathian parts of the Austrian Empire, which are the most mountainous districts of the Empire, is known to be greater than in other portions. But satisfactory data are wanting for more detailed remarks upon this subject.

If deaf-mutism is more prevalent in portions of country characterized by a marked elevation of surface, the reverse of this proposition should also be true, and we should expect to find countries whose physical aspect is entirely different more free from the privation than those previously mentioned. That this is the case is exhibited by the following facts. Belgium has a proportion of one in 2291; Saxony, one in 2180; Denmark, one in 1942; Holland, one in 2000. From sources where means of information are scanty, we cannot expect to throw much light upon this question; but if the test should apply with equal force to all countries, we should naturally infer that Italy and Turkey must be countries where deaf-dumbness is more prevalent, while Russia in Europe must be comparatively exempt. The countries of Asia, and especially the Chinese

Empire and Asiatic Russia, would be interesting subjects of study in this connection, on account of their numerous and lofty mountain-chains.

Perhaps in warm countries the ratio may be less than in those that are bleak and cheerless; and this may account for occasional paucity of deaf-mute population in mountainous regions; but of this we have not as yet sufficient facts to establish any positive speculations. More statistical returns, indeed, are needed to confirm the apparent coincidences of which we have spoken, and a more thorough knowledge of local phenomena than we at present possess. If any theory is to be deduced from the facts we have furnished from geographical sources, it can scarcely receive any decided confirmation until deaf-mute statistics are more perfectly recorded.

2. That *race* governs, to a certain extent, the distribution of the deaf and dumb, is exhibited in our own census-tables, more especially. We have scarcely any other means, indeed, of gaining knowledge upon this point, the contrast furnished by the white and colored population being very great, and information from other sources being deficient. Could we obtain statistics in the native country of the genuine African, we might discover by reliable data whether race alone operated, or whether locality and race combined to produce certain results We have no census of the Chinese population, nor of any of the Asiatic countries.

The colored race, it is satisfactorily shown, is less disposed to deaf-mutism than the white. The following table exhibits substantial grounds for the truth of this assertion:—

INFLUENCE OF RACE.

(*U. S. Census*, 1850.)

| | 1830. | 1840. | 1850. |
|---|---|---|---|
| Number of white deaf-mutes | 5363 | 6684 | 9136 |
| " " free colored " } " " slave " | 743 | 981 | 136 / 531 |
| White population | 10,537,378 | 14,195,695 | 19,553,068 |
| Free col'd " } Slave " | 2,328,642 | 2,873,758 | 434,495 / 3,204,313 |
| Proportion of white deaf-mutes to white population | 1 : 1964 | 1 : 2123 | 1 : 2140 |
| Proportion of free colored to free colored population } " " slave " slave " | 1 : 3134 | 1 : 2929 | 1 : 3194 / 1 : 6034 |

3. In regard to the *duration of life* of the deaf and dumb, and the age of greatest prevalence of deaf-dumbness, our census-tables are remarkably deficient, and we are compelled to cite results obtained in other countries; England, for example.

The numbers of the deaf-mute population are greatest between the ages of five and twenty-five; and as the age advances the numbers diminish. With the blind it is different; the proportions go on increasing from infancy to old age, and very rapidly in the latter years of life. Cases of blindness are comparatively rare at birth; and we know that causes very frequently operate in old age to produce blindness. Deaf-dumbness, on the other hand, is either congenital, or, as often happens, it is acquired as a sequence of those terrible visitations of malignant disease, from which young children suffer so disastrously.

In England and Wales the proportions of deaf-dumb and blind to the general population at particular ages are furnished in the following table:

In every 100,000 of the population there are—

| Years of age. | Deaf-Mutes. | Blind. | Years of Age. | Deaf-mutes. | Blind. |
|---|---|---|---|---|---|
| 0 | 42 | 40 | 45 | 100 | 280 |
| 5 | 159 | 59 | 55 | 98 | 515 |
| 10 | 167 | 73 | 65 | 89 | 1177 |
| 15 | 132 | 90 | 75 | 93 | 2556 |
| 25 | 115 | 107 | 85 and upwards, | 58 | 5672 |
| 35 | 107 | 163 | | | |

In regard to the comparative duration of life in the deaf and dumb and the blind, the same census gives this result:—

Of 12,553 deaf-mutes in Great Britain in 1851, only 783, or $6\frac{1}{5}$ per cent., arrived at the age of sixty.

Of 21,487 blind, 10,102, or 47 per cent., were at ages ranging from sixty upwards.

In this country we have a "Table of the Ages of White and Free Colored, Deaf and Dumb and Blind, in ten States in 1850," from which we glean the numbers of each class seventy years of age and upwards.

| States. | Deaf-mutes. | Blind. | States. | Deaf-mutes. | Blind. |
|---|---|---|---|---|---|
| Virginia, | 11 | 165 | Arkansas, | — | 11 |
| Vermont, | 2 | 43 | Ohio, | 8 | 140 |
| S. Carolina, | 1 | 45 | Michigan, | 2 | 21 |
| Louisiana, | 3 | 11 | Wisconsin, | 1 | 7 |
| Tennessee, | 3 | 100 | Iowa, | — | 7 |

In the States above-mentioned, there were—

| | Deaf-mutes. | Blind. |
|---|---|---|
| Under ten years of age . . . . . . | 481 | 173 |
| Ten, and under thirty . . . . . . . | 1343 | 585 |
| Thirty, " seventy . . . . . . | 681 | 917 |
| Seventy, and upwards . . . . . . | 31 | 550 |

If this be an accurate table, the number of deaf-mutes, at ages ranging from seventy years and upwards, is about one forty-third as great as between the ages of ten and thirty; while with the blind, the number is much the same at both periods.

4. The following table exhibits the number of deaf and dumb in each State of the Union, and their proportion to the general population. We have already seen that the colored race is less disposed to deaf-dumbness than the white, but no distinction is made in the table, the aggregate deaf-mute population in each State being taken into consideration.

*Table exhibiting the Aggregate Deaf and Dumb Population of the United States.* (*Census* 1850.)

| State. | | Deaf-Mute Population. | Population. | Proportion to Population. |
|---|---|---|---|---|
| Alabama, | | 210 | 426,514 | 1 : 2031 |
| Arkansas, | | 84 | 162,189 | 1 : 1930 |
| California, | | 7 | 91,635 | 1 : 13,090 |
| Columbia, D. of | | 19 | 37,941 | 1 : 1996 |
| Connecticut, | | 404 | 363,099 | 1 : 898 |
| Delaware, | | 54 | 71,169 | 1 : 1317 |
| Florida, | | 24 | 47,203 | 1 : 1966 |
| Georgia, | | 266 | 521,572 | 1 : 1960 |
| Illinois, | | 356 | 846,034 | 1 : 2376 |
| Indiana, | | 537 | 977,154 | 1 : 1819 |
| Iowa, | | 59 | 191,881 | 1 : 3252 |
| Kentucky, | | 563 | 761,413 | 1 : 1352 |
| Louisiana, | | 117 | 255,491 | 1 : 2183 |
| Maine, | | 266 | 581,813 | 1 : 2187 |
| Maryland, | | 261 | 417,943 | 1 : 1601 |
| Massachusetts, | | 358 | 985,450 | 1 : 2696 |
| Michigan, | | 125 | 395,071 | 1 : 3160 |
| Mississippi, | | 107 | 295,718 | 1 : 2763 |
| Missouri, | | 282 | 592,004 | 1 : 2099 |
| New Hampshire, | | 162 | 317,456 | 1 : 1959 |
| New Jersey, | | 189 | 465,509 | 1 : 2463 |
| New York, | | 1263 | 3,048,325 | 1 : 2413 |
| North Carolina, | | 471 | 553,028 | 1 : 1174 |
| Ohio, | | 915 | 1,955,050 | 1 : 2136 |
| Pennsylvania, | | 1145 | 2,258,160 | 1 : 1972 |
| Rhode Island, | | 65 | 143,875 | 1 : 2212 |
| South Carolina, | | 165 | 274,563 | 1 : 1664 |
| Tennessee, | | 377 | 756,836 | 1 : 2007 |
| Texas, | | 59 | 154,034 | 1 : 2610 |
| Vermont, | | 148 | 313,402 | 1 : 2117 |
| Virginia, | | 642 | 894,800 | 1 : 1393 |
| Wisconsin, | | 69 | 304,756 | 1 : 4416 |
| Minnesota, | Territories. | —— | 6,038 | —— |
| New Mexico, | | 34 | 61,525 | 1 : 1809 |
| Oregon, | | —— | 13,087 | —— |
| Utah, | | —— | 11,330 | —— |
| Total. | | 9803 | 23,191,876 | 1 : 2365 |

Dr. Harvey P. Peet, of the New York Institution for the Instruction of the Deaf and Dumb, has arranged the census of deaf-mutes, according to geographical divisions, so as to exhibit the relative proportions of white deaf-mutes to the white population.

| | |
|---|---|
| New England States - - - - - - - - - - | 1 in 1799 |
| Four Middle States - - - - - - - - - - - - | 1 in 2125 |
| Six N.W. States—Ohio, Indiana, Illinois, Iowa, Michigan, Wisconsin | 1 in 2160 |
| Five Southern States—Maryland, Virginia, North Carolina, South Carolina, Georgia, and D.C. - - - - - - - - - | 1 in 1821 |
| Eight S.W. States—Florida, Alabama, Mississippi, Louisiana, Arkansas, Tennessee, Kentucky, Missouri - - - - - - - | 1 in 2220 |
| Texas and New Mexico - - - - - - - - | 1 in 2800 |

The census returns show a greater amount of deaf-mutism among the native than among the foreign population. But there are several points to be taken into consideration, that may partially explain this. We do not know what proportion were born deaf and dumb, or how many became so in the district in which they were registered; nor do we know how many emigrated into the district who were at the time confirmed deaf-mutes. The following table, prepared also by the Principal of the N. Y. Institution, (Dr. Peet;) who is one of the most enlightened workers in the cause in which he has been so long engaged, exhibits the relative proportions among the foreign and native population, including white and free colored, but excluding slaves:—

*Proportion of Deaf-Mutes in the Native and Foreign Population.*

| States. | Native Population. | Native Deaf-Mutes. | Proportion to Population. | Foreign Population. | Foreign Deaf-Mutes. | Proportion to Foreign Population. |
|---|---|---|---|---|---|---|
| New England, | 2,423,223 | 1266 | 1 : 1914 | 299,340 | 66 | 1 : 4535 |
| New York, | 2,439,296 | 1168 | 1 : 2088 | 651,801 | 129 | 1 : 5053 |
| New Jersey, | 430,441 | 191 | 1 : 2253 | 58,364 | 12 | 1 : 4862 |
| Pennsylvania, | 2,014,619 | 908 | 1 : 2218 | 294,871 | 96 | 1 : 3071 |
| Seven N.W. States, | 4,656,158 | 2207 | 1 : 2110 | 638,784 | 195 | 1 : 3276 |
| Slave States N. of 35° | 3,642,248 | 2084 | 1 : 1748 | 119,730 | 23 | 1 : 5206 |
| " " S. " " | 1,892,565 | 714 | 1 : 2650 | 113,109 | 17 | 1 : 6653 |
| Total, | 17,498,550 | 8538 | 1 : 2049 | 2,175,999 | 538 | 1 : 4044 |

The subject of deaf-dumbness in the United States naturally suggests an inquiry into the past and present history of institutions charitably devoted to the instruction and care of those suffering from the deprivation. It is unnecessary to dwell upon details of modes of instruction, or to sketch at any length the rise and progress of deaf and dumb asylums; although it may be interesting to show how the benevolent wishes of the founders of deaf-mute instruction in this country have been satisfactorily accomplished, and how greatly the number of institutions has augmented since that time.

A father's solicitude for the welfare of his daughter, who had been deprived of the faculties of hearing and speaking by a violent attack of epidemic fever, then raging in Hartford, Connecticut, led to the establishment of the first school for the instruction of deaf-mutes in America, in the year 1817. In the previous year, legislative aid had been given in furtherance of such an object, and this is the first example of a benevolent legislative interposition in behalf of this afflicted class in America. To Gallaudet, under whose fostering care the institution increased rapidly in numbers and usefulness, is due the credit of founding in the United States a system which is now universally adopted throughout the country, and which has contributed so much to the amelioration of the condition of the deaf and dumb.

Other States rapidly followed the example thus set by the State of Connecticut; and twenty-one institutions have been founded in the following States, viz.:—Connecticut, New York, Pennsylvania, Kentucky, Ohio, Virginia, Indiana, North Carolina, Tennessee, Illinois, Georgia, South Carolina, Missouri, Wisconsin, Louisiana, Michigan, Iowa, Mississippi, Texas, District of Columbia, and Alabama.

From the latest sources of information in our possession, the condition of such institutions may be tabulated as follows:—

*Deaf and Dumb Institutions in the United States.*

| | Institution. | Location. | Date of Opening. | No. of Pupils. | According to Report for Year. |
|---|---|---|---|---|---|
| 1 | Connecticut, | Hartford, | 1817 | 252 | 1857 |
| 2 | New York, | New York, | 1818 | 302 | 1857 |
| 3 | Pennsylvania, | Philadelphia, | 1820 | 174 | 1857 |
| 4 | Kentucky, | Danville, | 1823 | 75 | 1857 |
| 5 | Ohio, | Columbus, | 1829 | 157 | 1857 |
| 6 | Virginia, | Staunton, | 1839 | 71 | 1854–5 |
| 7 | Indiana, | Indianapolis, | 1844 | 160 | 1857 |
| 8 | North Carolina, | Raleigh, | 1845 | 45 | 1850 |
| 9 | Tennessee, | Knoxville, | 1845 | 80 | 1856–7 |
| 10 | Illinois, | Jacksonville, | 1846 | 115 | 1856 |
| 11 | Georgia, | Cave Spring, | 1846 | 45 | 1856 |
| 12 | South Carolina, | Cedar Spring, | 1849 | 21 | 1856 |
| 13 | Missouri, | Fulton, | 1851 | 84 | 1856 |
| 14 | Wisconsin, | Delavan, | 1852 | 57 | 1857 |
| 15 | Louisiana, | Baton Rouge, | 1852 | 40 | 1856 |
| 16 | Michigan, | Flint, | 1854 | 57 | 1857 |
| 17 | Iowa, | Iowa City, | 1855 | 50 | 1857 |
| 18 | Mississippi, | Jackson, | 1856 | 21 | 1857 |
| 19 | Texas, | Austin, | 1857 | 11 | 1857 |
| 20 | District of Columbia, | Washington, | 1857 | — | —— |
| 21 | Alabama,* | | | | |

* Suspended in 1854–5.

5. Europe took the initiatory steps in the education of deaf-mutes nearly a century ago. In the year 1760, through the benevolent exertions of the Abbé de l'Epée, who may be considered the founder of institutions for the deaf and dumb, a school was organized in Paris, which for thirty years remained a private establishment. In 1791 the royal patronage of Louis XVI. was bestowed upon it, and it became a national Institution, under the care of the Abbé Sicard, who had received the advantages of L'Epée's able instruction, and who had been occupied as the presiding officer of an institution at Bordeaux previously to his removal to Paris.

The second school in Europe was established in Edinburgh, in 1764, by Braidwood. Then followed asylums in Leipzic, Bordeaux, London, etc., and their number rapidly increased, until, at the present day, we find them in almost all the European countries.

The following table gives all the information it has been practicable to obtain in regard to the past or present condition of those institutions. It is necessarily imperfect from the want of the proper materials for its compilation. It is modified from statistics in the N. Y. Report for 1851, as copied into the Report of the Kentucky Institution; later returns having been introduced to bring the table as much as possible up to recent times.

| Country. | No. of Institutions. | No. of Pupils. | Date of Information. | Location. |
|---|---|---|---|---|
| Austria, | 11 | 643 | 1851 | Vienna, Lintz, Brunn, Prague, Waitzen, Lemberg, Gratz, Halle, Gorz, Milan, Pressburg. |
| Bavaria, | 10 | 200 | 1835-38 | Munich, Bayreuth, Bamberg, Anspach, Wurtzburg, Nuremberg, Frankenthal, Dillengen, Straubing, Altdorf. |
| Belgium and Holland, | 14 | 539 | 1844-56 | Brussels, Ghent, Liege, Bruges, Mooreslade, Mons, Tournay, Namur, Maesyck, Antwerp, Groningen, Herlaar. |
| Denmark, Sweden, and Norway, | 5 | 296 | 1839-44 | Copenhagen, Schleswig, Stockholm, Drontheim, Christiania. |
| France, | 44 | 1542 | 1846-56 | Paris, Bordeaux, Marseilles, Toulouse, Albi, Le Puy, St. Etienne, Lyons, Grenoble, Vizille, Bourg, Sisteron, Clermont-Ferrand, Chaumont, Rodez, Aurillac, Poitiers, Loudun, Pont-Achard, Nantes, Auray, Laval, Angers, |

| Country. | No. of Institutions. | No. of Pupils. | Date of Information. | Location. |
|---|---|---|---|---|
| | | | | Nogent-le-Rotrou, Lamballe, Caen, Rouen, Besançon, Orleans, Strasburg, Nancy, St. Medard-les-Soiss, Arras, Lille, Vericelle, Villedieu, Pont l'Abbé. |
| German Free Cities, | 4 | 42 | 1844 | Frankfort, Hamburg, Bremen, Lubeck. |
| Great Britain and Ireland, | 23 | 1593 | 1851–56 | London, Birmingham, Manchester, Doncaster, Liverpool, Bristol, Exeter, Newcastle, Rugby, Bath, Brighton, Edinburgh, Glasgow, Dundee, Aberdeen, Dublin, Belfast, Cork, Swansea. |
| Italy, | 11 | 379 | 1834–51 | Rome, Naples, Sienna, Genoa, Turin, Modena, Milan, Villanuova, Verona, Ferrara, Bologna. |
| Prussia, | 25 | 853 | 1855 | Berlin, Stettin, Stralsund, Konigsberg, Angerburg, Marienburg, Posen, Breslau, Leignitz, Ratibor, Weissenfels, Erfurt, Halberstadt, Soest, Buren, Cologne, Moers, Kempten, Langenhorst, Halle, Aix-la-Chapelle, Braunsberg, Petershagen, Bruhl, Neuwied. |
| Russia and Poland, | 3 | 163 | 1838–50 | St. Petersburg, Warsaw, Odessa. |
| Saxony, Hanover, and rest of Germany, | 18 | 500 | 1838–48 | Leipzig, Dresden, Hildesheim, Weimar, Eisnach, Freiberg, Bansheim, Homberg, Camberg, Bruchoof, Brunswick, Wildeshausen, Habstahl, Emden, Altenburg, Coburg, Ilsfeld, Klausthal. |
| Spain and Portugal, | 3 | | | |
| Switzerland, | 10 | 256 | ——— | Geneva, Iverdon, Brunnader, Frienisburg, Einsiedelm, Zurich, Werden-Stein, Zofingen, Aarau, Riehen. |
| Wurtemberg and Baden, | 6 | 191 | 1844 | Gmund, Esslingen, Viennden, Tubingen, Wilhelmsdorf, Pfortsheim. |

The total number of European institutions given above is 187, with 7197 pupils. If it was possible to obtain more precise information in regard to these countries, the following general estimate would doubtless be found to be nearer the truth:—

Total number of European institutions - - - 220
" " " Pupils - - - - - - 10,000

Add to these the United States institutions, 20 or more in number, with nearly 2000 pupils, and a few scattered establishments elsewhere, and we shall find that there are at least 250 institutions in the world devoted to the same charitable purposes, and having under their care 14,000 or 15,000 pupils.

A probable estimate of the number of deaf and dumb in Europe is here given, prepared with a careful regard to accuracy, and based upon the most reliable statistical returns from most of the countries mentioned.

*Estimate of the Deaf-Mute Population of Europe.*

| Country. | Population. | Number of Deaf-Mutes. | Proportion to Population. | Date of Enumeration of Population. |
|---|---|---|---|---|
| Austria, | 38,088,400 | 24,030 | 1 : 1585 | 1852 |
| Bavaria, | 4,559,452 | 2922 | 1 : 1560 | 1852 |
| Belgium, | 4,426,202 | 1931 | 1 : 2291 | 1850 |
| Brunswick, | 271,208 | 192 | 1 : 1412 | 1852 |
| Church, States of, | 3,006,771 | 1897 | 1 : 1585 | 1850 |
| Denmark, | 2,296,597 | 1182 | 1 : 1942 | 1850 |
| France, | 35,783,170 | 29,512 | 1 : 1212 | 1852 |
| Germany, (except larger States,) | 6,088,737 | 3928 | 1 : 1550 | 1850–2 |
| Great Britain, | 21,121,967 | 12,553 | 1 : 1682 | 1851 |
| Greece, | 1,002,112 | 625 | 1 : 1600 | 1852 |
| Hanover, | 1,819,253 | 1230 | 1 : 1479 | 1852 |
| Holland, | 3,962,290 | 1981 | 1 : 2000 | 1853 |
| Ionian Islands, | 230,000 | 150 | 1 : 1600 | 1852 |
| Ireland, | 6,553,210 | 4151 | 1 : 1579 | 1851 |
| Modena, | 586,458 | 390 | 1 : 1500 | 1850 |
| Naples, (including Sicily,) | 8,704,472 | 5440 | 1 : 1600 | 1851 |
| Norway, | 1,328,271 | 1328 | 1 : 1000 | 1845 |
| Parma, | 507,881 | 317 | 1 : 1600 | 1853 |
| Portugal, | 3,471,203 | 2190 | 1 : 1585 | 1850 |
| Prussia, | 16,935,420 | 12,630 | 1 : 1340 | 1852 |
| Russia, | 60,098,812 | 37,917 | 1 : 1585 | 1851 |
| Sardinia, | 5,090,245 | 7272 | 1 : 700 | 1852 |
| Saxony, | 1,987,832 | 911 | 1 : 2180 | 1852 |
| Spain, | 13,936,218 | 8792 | 1 : 1585 | 1849 |
| Sweden, | 3,482,541 | 2279 | 1 : 1528 | 1850 |
| Switzerland, | 2,390,116 | 4751 | 1 : 503 | 1850 |
| Turkey, | 15,500,000 | 9687 | 1 : 1600 | 1844 |
| Tuscany, | 1,778,021 | 819 | 1 : 2171 | 1852 |
| Wurtemberg, | 1,733,263 | 1240 | 1 : 1400 | 1852 |
| Total for Europe, | 266,740,122 | 181,077 | 1 : 1473 | .... |

Of many parts of Continental Europe, and especially of the semi-civilized or barbarous countries of Asia and Africa, our information must necessarily be defective, no census of deaf-mutes having been taken at all, or, at any rate, not for many years, although we may have independent censuses of the general population. In such cases ratios furnished by former statistical tables have contributed to our present estimate of the number of deaf-mutes in a few of the countries named, and the proportion of deaf and dumb to the population in contiguous countries similarly situated has served for a guide in a small number of cases.

Tables have been published to show the proportion of deaf-mutes to the population of Europe, and these are, doubtless, not very far from the truth. Our own estimate corresponds very closely with that of Dr. Schmalz, of Dresden, who, in a statistical work upon this class, places the number of deaf and dumb at 145,131 in a general population of 214,000,000, a proportion of 1 in 1474. Another estimate by Dr. Lachman exhibits a greater amount of deaf-dumbness in Europe than we are induced to believe can probably exist, the proportion fixed upon by him being 1 in 1356. The difficulty of deciding, until each country establishes some means of obtaining authentic information, is very great, and conjecture must, in the mean time, supply what is at present inaccessible in any other way.

So also in regard to countries in which no census has ever been taken we must wait for the future to furnish the proper materials for comparison, unless, by conjectural estimates, we supply the deficiency. It is difficult to form a correct idea of the total deaf-mute population of the world, but the following summary may be an approximation:—

*Estimate of the Deaf-Mute Population of the World.*

| | Population. | Deaf-Mutes. |
|---|---|---|
| Europe | 266,740,122 | 181,077 |
| United States | 23,191,876 | 9803 |
| North America, South America | 35,000,000 | 20,000 |
| Asia, Africa, etc. | 600,000,000 | 375,000 |
| | 924,931,998 | 585,880 |

There are probably 600,000 deaf-mutes in the various countries of the world, or 1 in every 1550 or 1600 of the population.

The difficulty of deciding in early life whether a child is a deaf-mute will always be an insurmountable obstacle to a satisfactory estimate of the number of deaf-mutes in earliest infancy; but this is not the only difficulty. In cases of deafness uncomplicated with dumbness, of insanity or idiocy combined with deaf-mutism, and of dumbness unassociated with deafness, the ignorant and unscientific collector of statistics distributes the cases as he thinks fit, without any regard to the importance of minute details. Perhaps the same confusion may exist in some States from carelessness in regard to the separation of the statistics of the white population from the colored. So much doubt was entertained in regard to the censuses of 1830 and 1840 that a memorial was sent to Congress protesting against their publication. Assuming, however, that this is not a very serious source of error, can it be possible that a country like England has, in proportion to her population, so many more deaf-mutes than the United States? The ratio in Great Britain is 1 : 1670, while our own ratio of white deaf-mutes is 1 : 2140.

Perhaps the most interesting questions connected with deaf-dumbness are those which relate to the *causes* operating to produce it, whether such cases be congenital or acquired subsequently to birth; and the proportion existing between the two classes. These are subjects that have long excited attention with the philanthropist, and must always possess profound interest with the medical philosopher.

I. *The Causes of Congenital Cases* are family predisposition in some, marriages of consanguinity in others, and in others again, the influence of locality.

1. *Family Predisposition.*—Several instances of a curious and interesting nature in favor of the transmission of the infirmity through families are furnished by Mr. Wilde* from personal observation in Ireland. He gives an instance in the County of Cavan of the transmission of the disease, in direct descent, for three generations, the grandfather, the father, and four of the present family being all deaf and dumb. In the County of Limerick was found a mute with five paternal first cousins, and also a maternal second and a third cousin, all deaf and dumb. In the case of a mute individual in the County of Down, two grandaunts, two granduncles, and two cousins, on the mother's side, were mute. In one, in the County of Leitrim, two granduncles, and an uncle and aunt, by the father's side, were mute; and in Belfast was found a mute who had an uncle and aunt on the father's side, and an uncle and aunt on the mother's side, all deaf and dumb. In the County of Kerry two mute children in the same family had a grandaunt, an aunt, and a cousin, by the mother's side, deaf and dumb; and in the County of Fermanagh, where two mute children occurred in the same family, their three grandaunts and a granduncle, by the father's side, had been deaf and dumb. "*Uncles, aunts,*

---

* Wilde, William R., Practical Observations on Aural Surgery and the Nature and Treatment of Diseases of the Ear. Philadelphia, 1853. Appendix. Deaf-Dumbness, p. 444.

*and cousins, are the relatives of existing mutes who have most frequently exhibited the same disease.*"

The Irish census also furnishes 281 cases exhibiting the result of family predisposition or peculiarity in the production of congenital mutism, or of persons born in families, some of the previous members or collateral branches of which were mute, the disease being transmitted by the father's side in 149 cases, and by the mother's in 132. In five instances the grandfather was deaf and dumb; in three, the grandmother; in eighteen, the granduncles; in eleven, the grandaunts; in three, the father; in one, the mother; in forty-six, the *uncles;* in twenty, the *aunts;* and in 176, the *cousins*, had been deaf and dumb.

The report of the Hartford Institution for 1857 conveys additional evidence of the same kind. In a general summary of the causes, etc., of acquired deaf-mutism in all the pupils that had ever received the advantages of instruction in that school there are the following items in regard to deaf-mutes and their relatives:—thirteen pupils in five different families had one deaf and dumb parent; ten had both parents deaf and dumb in five different families; two had a deaf and dumb mother and grandfather; one had a deaf and dumb father and grandfather; one had deaf and dumb parents and a grandfather; one had a deaf and dumb great-grandfather; three married couples have each one deaf and dumb child; three couples have three deaf and dumb children each; one couple have two deaf and dumb children; one deaf and dumb husband with a hearing wife had three deaf and dumb children; one deaf and dumb wife with a hearing husband has one such child; twelve have each an uncle or aunt deaf and dumb; one has one uncle and one aunt; one has two uncles deaf and dumb; twenty-one have each one deaf and dumb cousin; six have two cousins; three have each three cousins; three have each four cousins; one has five cousins; one has six cousins; twelve have each one relative more remote than cousin; five have two; three have three; three have four; one has five; one has six; one has eleven; and one has fourteen such relatives.

Interesting facts of the same kind are furnished by Dr. Peet in the valuable report of the New York Institution for 1853. Cases are cited by him which bear conclusive testimony to the tendency of deaf-mutism to be diffused in families. "In Kentucky there is a family in which there are fourteen deaf-mutes, viz.: a father and eight children, (six males and two females,) all mutes; a father, mother, and child, (female,) all mutes, and two cousins." A summary of cases of direct inheritance from one or both parents, affords satisfactory evidence that deaf-dumbness may occur in this way, although such cases must be rare.

Cases are given by Dr. Peet of a deaf-mute son of a deaf-mute father, a deaf-mute son of a deaf-mute mother, and a deaf-mute daughter of a deaf-mute mother; and in summing up the various accounts of family predisposition in different countries, he remarks: "If these are all the cases that exist in countries in which inquiries have been made, it will follow that not one in a thousand of the deaf and dumb inherited their misfortune from a parent or known ancestor."

Curious rules applicable to this mode of transmission seem to be partially developed by statistics. It would appear that "this infirmity is more apt to be inherited by the sons of a deaf-mute father, and by the daughters of a deaf-mute mother," and it is a remarkable fact "that the hearing brothers and sisters of a deaf-mute not unfrequently have deaf-mute children."

But the occurrence of deaf-dumbness in families seems to be regulated by no fixed rules, although there are often significant facts having an apparent tendency toward obedience to a general law.

The subject of *deaf-mute marriages*, as a predisposing cause of congenital deaf-dumbness, is interesting, mainly because there seems to be a difference of sentiment as to whether they result in a transmission of the infirmity to the offspring. Attention has been paid to this branch of the inquiry of late years only; and while some authorities infer from statistics that the occurrence of congenital deaf-dumbness is more likely to be greater among children whose parents were both mutes, than among those whose father or mother were perfectly healthy, or one a hearing person and the other a mute, cases of hereditary transmission are so seldom known, that marriages between mutes are now frequently consummated without fear of detriment to the offspring.

Of the whole number of pupils of the Hartford Institution, from the time of its opening to the date of the last report, (1857,) one-third have been married, and three-fourths of such marriages have been between deaf-mutes. "Of the one hundred and forty-two families formed of our pupils who have married, there are deaf and dumb children in only nine of them, while in other families of six or eight children each, all can hear and speak. In conclusion of what we have to say on this head, we would remark, that while it would not be a matter of surprise if persons afflicted with congenital deafness should have deaf and dumb children, still it is far more probable that their children would hear and speak."

Information is still very deficient upon this point, and few works have as yet entered largely into the consideration of the question. Those attached to institutions,—who have more opportunities for studying such facts,—might often give valuable information upon points that are now wrapped in doubt, especially if, as in the case of the Hartford Asylum, they watch carefully the course of their pupils after leaving the institution.

The following extract from the *Liverpool Medico-Chirurgical Journal*, July, 1857, which we obtain from the "*Journal de la Physiologie de l'Homme et des Animaux*," edited by Dr. E. Brown-Séquard, January, 1858, gives a view of the subject so different from that of the Hartford Asylum, that it is deemed advisable to mention it. The article is on the hereditary transmission of deaf-dumbness, by Dr. Buxton; and the author declares that the probability of the existence of deaf-dumbness in children whose parents are both deaf-mutes, is seven times greater than in those who have but one deaf-mute parent. If a given number of children, 540 for example, have a deaf-mute father or mother, and the same number have both parents deaf-mute, there would be twenty-seven among the latter, while there would be but four among the former.

The experience of Dr. Peet is cited by the author of the above article, to prove the same facts; but in his *Annual Report* for 1854, we find the following passage: "It should be observed, that the marriages of deaf-mutes have been rare till within a few years, and hence, that we are not yet provided with a sufficient number of facts to warrant us in assigning positively the proportion of such marriages in which the children may be expected to inherit the infirmity of their parents. We can, however, show that it is much the most common for the children of deaf-mute parents to possess the faculties of which their parents were deprived. More than 190 of our former pupils are known to us to have been married, and only two couple, so far as we know, have deaf-mute children. * * * Of the two cases in which our former pupils have had deaf-mute children, in one the parents were, as we believe, both born deaf, and the mother was one of seven children born deaf out of fourteen. All their children, six in number, (three of each sex,) were born deaf." The passage referred to by Mr. Buxton is probably this: "The marriage of two deaf-mutes is more liable to produce deaf-mute children than the marriage of a deaf-mute with a hearing person, probably in the proportion of six or seven to one. Still the chance is small, except in a few remarkable families, where deaf-mutism seems developed in several branches as a family trait."

In a letter to my father, written in 1853, in answer to inquiries upon the subject,* Mr. A. B. Hutton, Principal of the Philadelphia Institution, makes the following remarks: "As to the propagation of the infirmity by deaf-mute parents, I would say that, from our experience, we have inferred that deaf-mute parents are no more liable to have deaf-mute children than other persons. We have heard of a number of marriages where all the children could hear and speak, with two or three rare ex-

* Dunglison's Human Physiology, vol. ii., p. 689; Phila., 1856.

ceptions. We have, at present, a mute child of deaf-mute parents, but the cases are few. In Europe it has been observed that the infirmity reappears in the third or fourth generation, and this seems to be partially confirmed by the observations of Hartford and New York."

Mr. Wilde* observes: "It is remarkable that while mutism is often manifest in several members of a family derived from a common stock, the defect is seldom transmitted direct from parents to children."

2. The subject of the *intermarriage of relatives*, as productive of affections of a deplorable nature has not, until lately, received the attention it merited. So much time is required to collect data for general laws upon this special point, that it can hardly be said that all have arrived at similar conclusions in regard to it. We know, however, as far as our observations have reached, that such alliances are very frequently most pernicious in their consequences to the offspring. If it were possible to make comparisons between an equal number of children, offspring of marriages of consanguinity and of marriages where no relationship of the parties existed, we might be more competent to form correct conclusions. While one authority seems to consider this a most important cause of deaf-mutism, and to think that "the question has been set at rest by the results of the Irish census," another cites negative testimony to cast a doubt upon the fact of deterioration of this kind occurring so frequently as is generally supposed.

The statistics of Ireland assuredly give as satisfactory a statement as could well be desired in proof of the existence of such active influences: 154 instances were collected, in which the parents were related in the degrees of first, second, or third cousins. The result of these intermarriages was nearly 100 cases—"eighty-six congenital and six acquired—of one mute in a family; four of these were dumb only; and four were dumb and idiotic. In thirty-four families, where the parents were related, two children were deaf and dumb, in only one instance of which the disease occurred after birth. There were fourteen instances where three mutes were born in families so circumstanced; and three where four in each family were deaf and dumb. The parents were also closely related in instances where six and seven in a family were similarly afflicted."†

Such testimony as is afforded by the careful observations of those who have made the subject a special study is too powerful to be resisted; and although we may have negative testimony of such marriages taking place without being attended with such unhappy results, our belief in this as one of the predisposing causes of deaf-mutism must remain unchanged. All who have opportunities afforded them for collecting facts bearing

* *Op. cit.*, p. 444. † Wilde, *Op. cit.*, p. 442.

upon the solution of these difficult questions, and all who are interested in the diffusion of a knowledge of the general laws that seem to regulate the existence or absence of deaf-dumbness, should give their attention to these points. They should examine the matter as a question not yet decided; and while they may be disposed to infer at the outset that marriages of consanguinity are often unfortunate in their effects, let them bear in mind that there are parts of the world where tribes are in the habit of marrying among themselves, and that evil consequences of the kind mentioned have not been noticed. From inquiries instituted in some portions of Europe, and in one village especially, where a quarter of the marriages were between first cousins, there seemed to be no prejudice existing against such alliances on the ground of degeneration in the offspring.*

Dr. Peet† shows, from the facts furnished by Mr. Wilde as the results of the Irish census, that of congenital cases about one in sixteen were the offspring of parents who were related within the degrees mentioned, but of the accidentally deaf, only about one in seventy; and that congenital deafness appeared at least four times, perhaps five times as often from a marriage between cousins, as from a marriage between persons not related. In other words, if in Ireland, in the general population, one child in 1700 is born deaf, of the children of cousins about one in 350 or 400 is born deaf. From the result of observations in the United States, he infers that if there is one child congenitally deaf in 3600, of the children of cousins there will be about one in 700.

3. Of *locality*, as a predisposing cause of the spread of deaf-dumbness, we have partially treated in the investigation of the prevalence of the infirmity in mountainous districts of country. But another point, elicited by censuses, is the fact of the greater proportion existing in rural districts as compared with cities. This is applicable only to congenital cases; epidemics of a fearful nature sometimes visit cities, and leave the country untouched; and deaf-dumbness follows naturally in the train, thus adding extensively to the number of cases arising subsequently to birth. Besides this, the nature of city life, in some of its worst phases, is such as to induce disease and suffering of the most unfortunate kind, and thus act as agents in the spread of deaf-mutism. It is in crowded portions of cities that we often see scrofulous cases or the most violent forms of epidemic scarlatina, and these are two important antecedents of deaf-dumbness.

The Belgian statistics exhibit the following figures in relation to the greater prevalence of deaf-mutism in country districts; as the majority

---

* Nottingham, Diseases of the Ear. Illustrated by Clinical Observations. 8vo. London, 1857.

† New York Report, 1854.

of such cases are congenital, it is more expedient to introduce the subject under the head of congenital causes.

| | |
|---|---|
| Number of deaf-mutes in cities - - - - - - | 373 |
| " " " country - - - - - | 1373 |
| Population in cities - - - - - - - - | 946,807 |
| " " country - - - - - - - - | 2,938,700 |
| Proportion of deaf-mutes to city population - - - | 1 in 2540 |
| " " country population - - | 1 in 2140 |

It is unfortunate that we are unable to furnish any statistics bearing upon this question from our last census returns, no trouble having been taken to separate cities and counties from each other. If the census of 1840 be taken as a guide, we may deduce some information as to the greater amount of deaf-dumbness in rural districts.

| | | |
|---|---|---|
| Massachusetts (except Boston) had - - - - | 42·5 | deaf-mutes in every 100,000 of population. |
| Boston had - - - - - | 17 | " " " " |
| New York State (except New York City) - - - - | 42·6 | " " " " |
| New York City - - - | 25·9 | " " " " |
| Pennsylvania (except Philadelphia) - - - - - | 43·9 | " " " " |
| Philadelphia - - - | 20·8 | " " " " |
| Maryland (except Baltimore) | 58 | " " " " |
| Baltimore - - - - | 30 | " " " " |

These statistics refer to the deaf and dumb as a class, and without any distinction of congenital and acquired cases. They prove, however, the general fact that, from various causes, the number in the country, as contrasted with the cities, is very large.

The Irish census gives us also an idea of the ratio existing between congenital and acquired cases in the city and country. " In the former class, the proportions were one in 2115 in the civic, and one in every 1670 in the rural, whereas in the latter the reverse obtained—the acquired being in the proportion of one in 9104 in the civic, and one in 13,107 in the rural."

II. *Causes of Acquired Deaf-Dumbness.*—It is difficult to classify all the causes of deaf-dumbness systematically. Each arrangement heretofore adopted has some peculiarity, and no uniform plan has been agreed upon, by which all the institutions of this country or of other countries could compare the results of their observations. The simplest mode of classification, according to Mr. Wilde,* is that which embraces affections

* *Op. cit.*, p. 456.

acting locally on the organ of hearing, diseases and accidents affecting the brain and nervous system, and unclassified causes. But this is a classification which, in a large number of cases, would embrace, under the head of "Unclassified," so many causes not allotted to the other heads, and so few of the tables hitherto prepared could be compared with it, that it is thought better in the present article to suggest what is believed to be a new arrangement, under which all the various causes can be satisfactorily comprehended.

The division is into heads, according as the causes of acquired deaf-dumbness are of pathological origin or are mechanical, a third division including causes whose nature has not been discovered. Examples of special diseases are given under different subdivisions, without any attempt to make the table sufficiently full to include all the multitude of causes that may operate.

*Classification of Causes of Acquired Deaf-Dumbness.*

I. PATHOLOGICAL CAUSES:
  1. Diseases of Respiratory and Circulatory Apparatus.
     Croup and Hooping-Cough.
     Catarrhal Affections.
     Pneumonia.
  2. Diseases of Digestive Apparatus.
  3. Affections of Nervous System and Organs of Sense.
     Paralysis and Apoplexy.
     Epilepsy, Chorea, Convulsions, etc.
     Encephalitis, etc.
     Sudden Emotions.
     Various Affections of Head, Neck, and Ears.
     Unclassified Nervous Affections.
  4. Diseases of Various Organs.
     Scarlatina.
     Measles.
     Smallpox.
     Fevers (unclassified.)
     Scrofula.
     Rheumatism, Dropsy, etc.
  5. Unclassified Pathological Causes.

II. MECHANICAL CAUSES:
  Falls, Injuries to Head, Ears, etc.
  Effects of Cold and Exposure.
  Immersion in Water.
  Burns.
  Unclassified Mechanical Causes.

III. UNKNOWN CAUSES.

In the "American Annals of the Deaf and Dumb" for April, 1856, there is an "abstract of seven hundred and eighty-seven cases, collected from the institutions for mutes at Paris, Copenhagen, Leipzic, Prague, Cologne, St. Petersburg, Dresden, Hamburg, and Modena, in Europe; and Hartford, New York, Philadelphia, and Columbus, in the United States." This is republished from the Eighteenth Report of the New York Institution. The general average of causes, as deduced from that table, is as follows:—

| | | |
|---|---|---|
| Pathological Causes | - - - - - - | 44 per cent. |
| Mechanical " | - - - - - - | 5 " " |
| Unknown " | - - - - - - | 51 " " |

This is a very unsatisfactory classification, on account of the evident want of accuracy in the statistics of causes in many of the institutions named; the number of unknown causes being so much larger than those which were ascertained.

The causes of deafness in Europe, in 1630 cases, have been arranged in a table* which, modified, gives the following result:—

| | | |
|---|---|---|
| Pathological Causes | - - - - - - | 71·5 per cent. |
| Mechanical " | - - - - - - | 9 " " |
| Unknown " | - - - - - - - | 19·5 " " |

This corresponds very closely with the experience of the Hartford Asylum in the case of its own pupils.

In 1314 cases recorded in the institutions of the United States, the proportion was found to be as follows:—

| | | |
|---|---|---|
| Pathological Causes | - - - - - - | 68 per cent. |
| Mechanical " | - - - - - - - | 5·3 " " |
| Unknown " | - - - - - - - | 26·7 " " |

Perhaps if all the causes were known, the difference between Europe and the United States in the general average would be but slight, whatever variation there might be in regard to special diseases.

The most frequent causes of deaf-dumbness in Europe and America are scarlatina, measles, and fevers of various kinds. In the United States, scarlatina is by far the most active exciting cause, while in Europe, if statistics be reliable, fevers seem to occupy about the same position as it holds with us. The relative numbers may be thus given:—

In every 1000 cases of deaf-mutes, there were of

| | | |
|---|---|---|
| Scarlatina - - - - | 80 in Europe, | 200 in the United States. |
| Measles - - - - | 34 " " | 43 " " |
| Fevers - - - - - - | 175 " " | 133 " " |

* Thirty-Fifth New York Report.

Whenever epidemics of scarlatina occur in any part of a country, the proportion of deaf-mutes to the population is likely to increase, and this has been markedly the case in our own country. Parts of Europe, too, suffer from its ravages, but it is in the United States that it forms a most alarming cause of deaf-dumbness. One-fifth of the whole number of cases are the results of violent attacks of scarlatina; fevers of all kinds being the next most important causes. In Europe about one-sixth of the cases have been preceded by severe fevers, which have resulted in a destruction or irreparable injury of the organs of audition. We rarely hear of towns or districts of country in which the disease of early infancy, scarlatina, is of so common occurrence as with us.

Dr. Peet thinks it is only of late years that scarlatina has been so great a scourge to our country, and he exhibits the following figures to confirm this view. In Philadelphia, "the whole mortality from this disease, from 1815 to 1829, inclusive, was 92. In 1830 there were 40 deaths by this disease; in 1831, 200; from 1831 to 1846, inclusive, 3391."* The number of cases of deaf-mutism admitted into the American institutions, preceded by scarlatina, has increased greatly since 1840. Of the pupils previous to that year, one case in forty-one was ascribed to scarlet fever; since that time, about one case in eight.

What proportion of cases of deaf-mutism are congenital, or, in other words, what is the ratio of acquired cases to congenital?

The experience of the Hartford Asylum, as recorded by its able principal, Rev. W. W. Turner, is here given:—

| | |
|---|---|
| Cases of congenital deafness | 542 |
| Hearing lost | 483 |
| Not ascertained | 51 |
| Not deaf | 5 |
| | 1081 |

The proportion deduced from this statement is as follows:—

| | |
|---|---|
| Congenital cases | 50 per cent. |
| Acquired | 44 " " |

A general summary of the two classes of cases, compiled from many of our American reports by Dr. Peet, gives the following result in 3855 cases:—

| | |
|---|---|
| Cases of congenital deafness | 1812 or 47 per cent. |
| " " acquired " | 1569 or 41 " " |
| Unknown | 474 |
| | 3855 |

* New York Report, 1854.

From the same source we learn several facts in regard to the two classes of cases in Europe. The American figures are here added to facilitate a comparison. It will be observed that the general ratio for Europe differs widely from this country, and that Germany has a ratio peculiar to itself.

| | Congenital. | Acquired. |
|---|---|---|
| Sum of European returns (except German schools) - | 400 | 100 |
| German institutions - - - - - - - - - | 95 | 100 |
| United States - - - - - - - - - - - | 116 | 100 |

In Ireland eighty per cent. were congenital, or a ratio of 400 congenital to 100 acquired; while in Belgium, there were 373 to every 100.

*Diseases Coexisting with Deaf-Dumbness.*—Belgium has, in one of her elaborate works, furnished a list of diseases which have been found to occur coexistently with deaf-mutism. It is valuable to the medical profession, and is here translated, as it is rarely that we meet with a summary of the kind. It is collected from various sources.

| | Deaf-Dumbness. | | |
|---|---|---|---|
| | Congenital. | Acquired. | Total. |
| Idiocy, or Mental Alienation - - | 78 | 12 | 90 |
| Paralysis - - - - - - - | 23 | 9 | 32 |
| Epilepsy - - - - - - | 12 | 4 | 16 |
| Blindness - - - - - - - | 3 | 2 | 5 |
| Loss of an Eye - - - - - | 6 | 2 | 8 |
| Weakness of Sight - - - - - | 1 | 3 | 4 |
| Asthma - - - - - - - | 2 | — | 2 |
| Rachitis - - - - - - - - | 3 | — | 3 |
| Gôitre - - - - - - - - | 6 | — | 6 |
| Hernia - - - - - - - - - | 2 | — | 2 |
| Diseases not specified - - - - | 7 | 4 | 11 |
| Lameness - - - - - - - | 6 | 2 | 8 |
| | 149 | 38 | 187 |

We learn, therefore, that where deaf-dumbness was complicated with other affections, about forty-eight per cent. were cases of mental defect of some kind, as idiocy or insanity, and about seventeen per cent. paralysis. The materials afforded, however, are too scanty for us to deduce any general laws.

*Proportion of the Sexes.*—The male sex among the deaf-mutes is largely in the majority, even in countries where there are more female than male inhabitants. This is a rule that obtains in almost every country. Great Britain has 121 male deaf-mutes to every 100 females, and in Scotland the inequality is somewhat greater, 125 males to 100 females. The congenital cases vary from the acquired upon this point, for we find in Ireland, for example, 100 males to 74·61 females in the former class, and 100 to 91·46 in the latter. The result in Europe, in 33,224 cases, was 14,573 females to 18,651 males, or 78·1 to 100, but no division is made into congenital and acquired cases. In the American schools, in 2754 cases, there were 771 male deaf-mutes to 665 females, among the congenital cases, or 100 males to 86 females; while there were 770 males to 548 females among the acquired cases, or 100 males to 71 females.*

*Post-Mortem Examinations of Deaf-Dumbness.*—In a large number of cases the true pathology cannot be studied during life, portions of the ear being affected which are not accessible to the observation of the physician. Post-mortem examinations, too, are exceedingly rare, so that much of what we know about the causes of deaf-mutism must be founded on conjecture. In congenital cases there is great reason for believing that the auditory nerve has not been fully developed, or that other portions of the apparatus have suffered some abnormal changes. The internal ear is doubtless the part most involved in congenital cases. In the case of "a deaf and dumb boy, five years and eight months old, who died of croup, on the 21st November, 1855," a post-mortem examination was made, and is thus given by Mr. Nottingham:†—

"This patient was clever and intelligent, and had commonly enjoyed good health, but from the earliest period of his life had been totally insensible to sound, and, of course, had never attained the power of speech; the head, the external ear, meatus, and membrana tympani, were well formed. The tympanum, on both sides, was found to be choked, if this expression may be employed, by a pale, grayish mass of apparently inspissated mucus; there was no other morbid appearance seen in the tympanic cavity, the ossicles, the membrana tympani, and the membranes of the fenestræ, being in normal condition. The eustachian tube was sound and pervious. The roots of the auditory nerve were exceedingly small—faint white lines—and the trunk of this nerve was still more remarkable for having less than the normal size. The small size of the lingual, or motor nerve of the tongue, was also remarkable; the glosso-pharyngeal nerve was also very small, and the thyroid body had less than its normal dimensions."

"In another case of congenital deaf-dumbness, in a girl, of nine years of age, who died with serous effusions into the ventricles of the brain, which followed an attack of scarlet fever, careful examination of every part of the auditory apparatus was made, but without the discovery of any deviation from the normal anatomical conditions, with the exception of an apparently small size of the trunk of the auditory nerve, which,

* Thirty-Fifth New York Report. † *Op. cit.*, p. 587.

having reached the bottom of the internal meatus, divided, and was distributed in the usual manner."*

In the *British and Foreign Medical Review*, vol. iii., p. 227, we find the translation of an article by Dr. Bergmann, first published in the *Hannoversche Annalen für die gesammte Heilkunde*, Band 1, Heft. 1, 1836, on the appearances found on dissection of a deaf and dumb person. The patient died of a fever, with pulmonary symptoms.

"The brain was in a normal condition, and its different parts exhibited no deviation from their usual structure. The external ear was of a natural conformation, and the meatus externus and membrana tympani presented no unusual appearances; but the membrane which lined the hollow of the tympanum was of a spongy, sarcomatous nature, and that which covered the bones of the internal ear was similarly affected, so that their motion might have been somewhat impeded in consequence. In the hollow of the tympanum of both sides there was a quantity of thickish mucus. A hard concretion, about the size of a mustard-seed, covered the fenestra rotunda on the left side. The semicircular canals were of a natural form, but degenerated internally; they seemed to have contained some fluid, but in very small and insufficient quantity. The thin, transparent lining membranes, (ductus semicirculares Scarpæ,) the ampullæ, and the nerves, could not be distinctly discovered on either side. The defects here were evidently of a nature to have interfered materially with the exercise of the function of the organ. They could not have been affected by the process of examination, for the appearances presented were precisely similar on both sides. The cochlea was found in its usual condition, and appeared to be properly supplied with nerves. Whether the defects visible in the semicircular canals were congenital or the result of disease, it was of course impossible to ascertain."

After enumerating all the varied causes of deaf-dumbness and the interesting statistical points which arise in connection with the subject, we ask ourselves, Is deaf-dumbness curable? Sad experience answers negatively in almost all cases, and the medical man laments his inability to supply deficiencies in what nature has neglected to accomplish, or to restore to healthy vigor that which disease has impaired or destroyed. There are few physicians who do not feel, when such cases present themselves to their notice, that their efforts must fail to alleviate the condition of their unfortunate patient, because they cannot remove the cause which has given rise to it.

But it may be asked, whether there are not some cases in which hope

* Nottingham, *op. cit.*, p. 589.

may be indulged, and an important sense be restored to normal action. Of course it is unnecessary to speak of congenital cases, for the most thorough knowledge of the healing art can never be sufficiently potent to supply defective, or to remedy a diseased, structure, which is far beyond the physician's reach. It is in acquired deaf-dumbness that we occasionally hear of cases in which benefit, it is affirmed, has been obtained. Too often these are the unfounded statements of itinerant aurists, who have no reputation to lose. When good effects have resulted from treatment in deaf-mutism, it has been in cases where the deafness has resulted from occlusion or obstruction of the Eustachian tube; and in strumous affections of the middle ear, the result of scarlatina. But, in almost all such, the benefit has been transient; and we are unhappily driven to the painful conclusion, that the deaf-mute must be left to the gloomy reflection that his malady is irremediable.

